# Effect of hyperbaric oxygen on BDNF-release and neuroprotection: Investigations with human mesenchymal stem cells and genetically modified NIH3T3 fibroblasts as putative cell therapeutics

**DOI:** 10.1371/journal.pone.0178182

**Published:** 2017-05-23

**Authors:** Jennifer Schulze, Odett Kaiser, Gerrit Paasche, Hans Lamm, Andreas Pich, Andrea Hoffmann, Thomas Lenarz, Athanasia Warnecke

**Affiliations:** 1 Department of Otorhinolaryngology, Head and Neck Surgery, Hannover Medical School, Hannover, Germany; 2 Cluster of Excellence “Hearing4all”, Hannover, Germany; 3 Core Facility Proteomics, Hannover Medical School, Hannover, Germany; 4 Department of Orthopaedic Surgery, Hannover Medical School, Hannover, Germany; University of Louisville, UNITED STATES

## Abstract

Hyperbaric oxygen therapy (HBOT) is a noninvasive widely applied treatment that increases the oxygen pressure in tissues. In cochlear implant (CI) research, intracochlear application of neurotrophic factors (NTFs) is able to improve survival of spiral ganglion neurons (SGN) after deafness. Cell-based delivery of NTFs such as brain-derived neurotrophic factor (BDNF) may be realized by cell-coating of the surface of the CI electrode. Human mesenchymal stem cells (MSC) secrete a variety of different neurotrophic factors and may be used for the development of a biohybrid electrode in order to release endogenously-derived neuroprotective factors for the protection of residual SGN and for a guided outgrowth of dendrites in the direction of the CI electrode. HBOT could be used to influence cell behaviour after transplantation to the inner ear. The aim of this study was to investigate the effect of HBOT on the proliferation, BDNF-release and secretion of neuroprotective factors. Thus, model cells (an immortalized fibroblast cell line (NIH3T3)–native and genetically modified) and MSCs were repeatedly (3 x – 10 x) exposed to 100% oxygen at different pressures. The effects of HBO on cell proliferation were investigated in relation to normoxic and normobaric conditions (NOR). Moreover, the neuroprotective and neuroregenerative effects of HBO-treated cells were analysed by cultivation of SGN in conditioned medium. Both, the genetically modified NIH3T3/BDNF and native NIH3T3 fibroblasts, showed a highly significant increased proliferation after five days of HBOT in comparison to normoxic controls. By contrast, the number of MSCs was decreased in MSCs treated with 2.0 bar of HBO. Treating SGN cultures with supernatants of fibroblasts and MSCs significantly increased the survival rate of SGN. HBO treatment did not influence (increase / reduce) this effect. Secretome analysis showed that HBO treatment altered the protein expression pattern in MSCs.

## Introduction

In the auditory system, neurotrophic factors (NTFs) are known to play important roles in the innervation of the inner ear. In particular, neurotrophin-3 (NT-3) and brain-derived neurotrophic factor (BDNF) are essential for the normal development and innervation of the inner ear, establishing precise connections between the hair cells and the auditory neurons, e.g., the spiral ganglion neurons (SGN) [1–3]. In the adult system, NT-3 is involved in the homeostasis of the inner ear, whereas the expression of BDNF is downregulated [4]. Hair cells and supporting cells release trophic factors that maintain the synaptic connections between hair cells and the afferent fibres of the SGN [5–7]. Furthermore, NT-3 delivered by supporting cells promotes recovery of cochlear function and regeneration of ribbon synapses [8]. A recent study has demonstrated a differential effect of NT-3 and BDNF: NT-3 increases regrowth of the afferent fibres whereas BDNF promotes survival of SGN [9]. Irreversible damage of auditory hair cells and degeneration of SGN result in sensorineural hearing loss (SNHL). In age-related hearing loss, a pronounced neuronal degeneration has been observed [10]. Recent research has shown that age and noise-induced damage is associated with permanent loss of ribbon synapses [11]. Profound to severe SNHL is usually treated with a cochlear implant (CI), which electrically stimulates residual SGN. The application of NTFs aims at preservation and regeneration of deprived SGN as well as the stabilization of the ribbon synapses. Several studies indicate that the delivery of NTFs like BDNF protects SGN and induces neurite outgrowth [12–14]. Overexpression of BDNF and NT-3 as a consequence of an adenoviral gene transfer induces local fibre regrowth [15]. However, NTFs like BDNF have a short serum half-life time [16]. Therefore, a long-term delivery of NTFs should be achieved. Different types of viral vectors have been used successfully for the long-term and stable delivery of NTFs [9,17,18]. In a previous study, a lentivirally modified cell line secreting BDNF was used to prevent SGN from degeneration and showed enhanced survival rates of SGN and neurite outgrowth *in vitro* and *in vivo* [14]. However, a clinically feasible method to influence cells transplanted to the inner ear is not available up to now. In this context, hyperbaric oxygen therapy (HBOT) seems to be a promising strategy. It utilizes the administration of 100% oxygen at greater pressures than normal atmospheric level and thus increases oxygen tension in blood and cochlea [19]. HBOT has been used for many years as a therapeutic modality of various diseases, including arterial gas embolism, carbon monoxide poisoning, decompression sickness, wound healing, sudden deafness, SNHL and acute noise trauma [20]. Thus, the therapeutic efficacy of HBOT may be enhanced by cell-based approaches for NTF delivery. Furthermore, HBOT may influence and modulate the behaviour of the transplanted cells.

Human mesenchymal stem cells (MSCs) represent a promising cell source for the treatment of inner ear damage. They can be transplanted in an autologous manner after isolation from the bone marrow or adipose tissue of the patient. In addition, they secrete NTFs including BDNF [21] and activate other cell types (e.g., glial cells) to secrete these NTFs [22]. Moreover, human MSCs have the capacity for self-renewal and differentiation in many different cell types. They show a high differentiation potential into mesenchymal tissue cells like osteoblasts, chondrocytes and adipocytes and can partly also differentiate into muscle cells and neurons *in vitro* [23,24]. Furthermore, they are characterized by the secretion of a variety of NTFs, promoting endogenous neuronal growth, inducing angiogenesis and neurogenesis, encouraging synaptic connection and axonal remyelination, decreasing apoptosis and regulating microglial activation [25]. The secreted trophic factors provide a supportive microenvironment for damaged tissue. The differentiation fate of MSCs depends on the environment within their particular niche. MSCs exist in perivascular niches in close association with blood vessels but nevertheless they are exposed to low oxygen pressure [26]. The oxygen level in human bone marrow amounted up to 7% oxygen [27]. The effect of HBOT on human MSCs is poorly understood, but several studies showed the beneficial effects of HBOT: promoting osteogenic differentiation by regulating the Wnt-signaling pathway [28], inducing placental growth factor expression [29], increasing the expression of fibroblast growth factor (FGF)-2 and the expression of Wnt-3 protein in neural stem cells [30]. Additionally, an induced vascular endothelial growth factor expression in endothelial cells of human umbilical vein was reported [31]. Moreover, HBOT was shown to increase the mobilization of stem / progenitor cells from the bone marrow *in vivo* by a nitric oxide-dependent mechanism [32]. MSCs play also an important role in wound healing processes because of their ability to migrate to injured sites while modulating the immune response. Therefore, they have high potential in human regenerative medicine, curing various diseases including neuronal injury or neurodegeneration (reviewed in [25]) with their therapeutic effects in neuronal settings being exerted by migration of MSCs to the injured region [32] and by secretion of trophic factors [33]. Concerning the development of a cell-covered CI electrode, a so-called biohybrid electrode, cells isolated from human bone marrow including MSCs seem to be a promising cell source. Recently, we have shown that the use of biohybrid electrodes is suitable in human neurosensory restoration ([34]). Autologous cells including MSCs may protect residual SGN as well as reduce foreign body reaction and inflammation. The survival of MSCs in the normal cochlea after injection in the scala tympani was already shown [35]. Moreover, it was demonstrated that MSCs differentiate *in vitro* into neurons expressing SGN-specific markers [36].

In the present *in vitro* study, NIH3T3 fibroblasts served as a model for the establishment of an adequate protocol for HBO treatment before human bone marrow-derived MSCs were tested as an autologous cell source for the development of a biohybrid electrode. The aim of the study was to investigate if HBOT as non-invasive clinical therapy for inner ear damage can influence the behavior of cells in terms of proliferation and release of neurotrophic factors in comparison to normoxic control cells.

## Materials and methods

### Ethics statement

For the isolation of MSCs, human bone marrow aspirates were obtained after approval by the institutional ethical committee of Hannover Medical School. Written informed consent was obtained from all donors. All personal information was made anonymous. The bone marrow was harvested by iliac crest aspiration during routine orthopaedic procedures from otherwise healthy persons.

The experiments were performed in accordance with the institutional guidelines for animal welfare of the Hannover Medical School following the standards described by the German “Law on Protecting Animals (Tierschutzgesetz) and with the European Directive 2010/63/EU for protection of animals used for experimental purposes. SGN were isolated from neonatal Sprague-Dawley rats, which were decapitated in accordance with the German animal welfare act. The euthanasia for our in vitro experiments is registered (no.: 2013/44) with the local authorities (Zentrales Tierlaboratorium, Laboratory Animal Science, Hannover Medical School, including an institutional animal care and use committee) and is reported on a regular basis as demanded by law. For exclusive sacrifice of animals for tissue analysis in research no further approval is needed if no other treatment is applied beforehand (§4). The rats were bred and born for research study purposes. A breeding stock was supplied by Charles River (Charles River, Wilmington, USA) and housed with their litters in the facilities of the licensed Institution of Laboratory Animal Science of the Hannover Medical School. To minimize the stress level for the neonatal rats, they were euthanized by decapitated prior to any experimentation by a licensed person.

### Fibroblast cell culture

Two different types of the murine fibroblast cell line NIH3T3 were used for the experiments: standard NIH3T3 cells (ATCC-Number: CRL-1658; denoted as: NIH3T3) as a control and genetically modified cells for the expression and secretion of human BDNF. According to the previously described protocol [37], a tetracycline-regulated plasmid (pLOX) was used as lentiviral vector for the generation of a doxycycline-regulated fibroblast cell line (NIH3T3) secreting BDNF as well as expressing green fluorescent protein (GFP) as a marker (denoted as NIH3T3/BDNF). NIH3T3 and NIH3T3/BDNF fibroblasts were cultured in Dulbecco’s Modified Eagle’s Medium (DMEM, FG0445, high glucose; Biochrom, Berlin, Germany) supplemented with 10% fetal calf serum (FCS, Biochrom) as well as 1% penicillin and streptomycin (Biochrom). Additionally, 1 μg/mL doxycycline (DOX) was added to the cultures of NIH3T3/BDNF fibroblasts for induction and maintenance of the promotor-regulated gene expression of BDNF.

### Mesenchymal stem cells

Human bone marrow-derived MSCs were isolated from human bone marrow aspirates of two different donors. Donor A was a 22-year old male and donor B was a 23-year old female. Mononuclear cells were isolated by density gradient centrifugation with a Biocoll gradient (Biochrom). Plastic-adherent cells were passaged before reaching confluence and were sub-cultured at a density of 2 x 10^3^ to 5 x 10^3^ cells/cm^2^ in Dulbecco´s Modified Eagle´s Medium (DMEM, FG0415, Biochrom) supplemented with 10% fetal calf serum (FCS, Thermo Scientific, Hyclone, Waltham, USA), 25 mM 4-(2-hydroxyethyl)-1-piperazineethanesulfonic acid (HEPES; Life Technologies), 100 U/mL penicillin (Biochrom), 100 mg/mL streptomycin (Biochrom) and 2 ng/mL human recombinant FGF2 (Peprotech, Hamburg, Germany). For all experiments, cells were used in passage 6. MSCs characteristics were confirmed randomly by flow cytometric analysis of cell surface molecules and by *in vitro* differentiation as described elsewhere [38,39].

### Hyperbaric oxygen treatment

Cells (fibroblasts and MSCs, respectively) were seeded in 48-well plates (Nunc, Langenselbold, Germany) with 2 x 10^3^ cells/cm^2^ and were cultured at 37°C and 5% CO_2_ for 24 hours. Prior to the first HBO treatment, the medium was exchanged in all cultures to set all conditions to zero. For hyperbaric oxygen (HBO) treatment, the plates were transferred into an experimental compression chamber, which was flushed with 100% oxygen for 30 sec and then pressurized to the respective level (1.0 bar, 1.5 bar or 2.0 bar) for 90 min at room temperature (RT) for five times (fibroblasts; Monday to Friday) and ten times (MSCs; Monday to Friday for two weeks). After HBO exposure, the chamber was slowly decompressed. Control cells (fibroblasts and MSCs) were incubated for 90 min at RT under normoxic and normobaric (NOR) conditions: ambient room air and atmospheric pressure. After HBO and NOR treatment, cells were incubated for 22.5 hours at standard conditions until the next treatment. One day post exposure (see Table 1), cells from all groups were counted and supernatants were collected, sterile filtered (0.22 μm, Millex-GV, Merck Millipore; Darmstadt, Germany) and stored at -80°C for further experiments. Differing from that, the cells of the fifth/tenth treatment were analysed after the weekend, i.e., after 72 hours (see Table 1). The medium of the MSCs was exchanged every three to four days (i.e., on Monday and Thursday) whereas the fibroblast experiments were performed for one week without changing the medium.

**Table 1:** Oxygen treatment scheme.

Number of treatments	1	2	3	4	5	6	7	8	9	10
**Counted after [hours]**	24	24	24	24	72	24	24	24	24	72
**Days in culture**	2	3	4	5	8	9	10	11	12	15
**NIH3T3**	X	X	X	X	X					
**NIH3T3/BDNF**	X	X	X	X	X					
**MSCs**	X	X	X	X	X	X	X	X	X	X

### Cell proliferation/cell counting

Cells were detached with a trypsin/EDTA solution (0.25%; Biochrom) by incubating for 5 min at 37°C and 5% CO_2_. Trypsin was inactivated by adding serum-containing medium and cells were resuspended. The number of viable cells was determined in duplicates by using a Fuchs Rosenthal chamber (Brand, Wertheim, Germany) for MSCs and a Neubauer chamber (Neubauer improved; Brand,) in combination with the trypan blue exclusion test (dilution 1:2; Sigma-Aldrich, Taufkirchen, Germany) for the fibroblasts. In each experiment, the same person performed the counting in order to rule out interindividual variations. Random controls counted by a second blinded examiner were not significantly different. The data for each time point were collected from triplicate wells. Each experiment was repeated three times and growth curves were plotted from acquired data (N = 3, n = 3). Additionally, cell morphology was checked every day by light microscopy and exemplary photographs were taken.

### Spiral ganglion cell culture

The neuroprotective effect was investigated by adding collected supernatants of HBO-treated cells (fibroblasts and MSCs, respectively) to freshly isolated SGN. Neonatal Sprague-Dawley rats of both sexes (postnatal day three to five) were used for preparing the primary cell culture. Isolated cochleae were microscopically dissected followed by enzymatic and mechanical dissociation of the spiral ganglia, which was performed according to the previously described protocol [40]. The viable cells were counted as described above by using a Neubauer chamber and trypan blue exclusion test. Before cell seeding, plates were coated with poly D/L-ornithine (0.1 mg/mL; Sigma-Aldrich) and laminin (0.01 mg/mL; natural from mouse, Life Technologies, Carlsbad, USA) as described in detail by Wefstaedt and colleagues (2005) [40]. The dissociated cells were seeded at a density of 1 x 10^4^ cells per well in a flat-bottom 96-well plate (Nunc). The SGN were cultivated in a mixture (1:1) of SGN medium and the supernatants collected from the HBO- and NOR-treated cells as well as different control conditions. The three supernatants of the same condition and experiment of the fibroblasts were pooled. SGN medium consisted of serum-free Panserin 401 (PAN Biotech, Aidenbach, Germany), supplemented with HEPES (25 mM; Life Technologies), glucose (6 mg/mL; Braun AG, Melsungen, Germany), penicillin (30 U/mL; Grünenthal GmbH, Aachen, Germany), N2-supplement (3 μg/mL; Life Technologies) and insulin (5 μg/mL; Sigma-Aldrich).

After a cultivation period of 48 h, cells were fixed with a 1:1 acetone (J. T. Baker, Deventer, Netherlands)/methanol (Roth, Karlsruhe, Germany) solution for 10 min and were washed with phosphate-buffered saline (PBS; PBS tablets, Gibco® by Life Technologies). In addition, a seeding control was fixed already after 4 h. A negative control (SGN in serum-deprived SGN medium), a positive control (SGN in SGN medium supplemented with 50 ng/mL BDNF) and a medium control (SGN in 1:1 fibroblast/MSC medium; containing FCS) were also included in every experiment.

### Survival rate and neurite length of SGN

The dissociated SGN cultures were mixed cultures containing other cell types aside from neurons, such as fibroblasts or glia cells. For identification of SGN, a neuron-specific staining was used. Acetone/methanol fixed cells were therefore stained with a mouse 200 kD neurofilament antibody (clone RT97; Leica Biosystems, Wetzlar, Germany), a secondary biotinylated anti-mouse antibody and ABC complex solution as described in detail previously using the Vectastain® Elite® ABC Kit [40]. The antibody complexes were visualized by the addition of diaminobenzidine (Peroxidase Substrate Kit DAB; Vector Laboratories Inc., Burlingame, USA). Surviving neurons were defined as neurofilament-positive cells which exhibit a neurite length of at least three cell soma diameters [12] and counted by using an inverted microscope (Olympus CKX41, Hamburg, Germany). The neuroprotective effect was evaluated by determining the survival rate which was calculated by relating the number of survived neurons to the mean seeding density (mean number of neurons in the seeding control) of the same plate.

To examine the neuroregenerative effect of the NIH3T3 fibroblasts, the five longest neurons in each field of view (one in the centre and four around the perimeter of the well) were imaged using a transmission light microscope (Olympus CKX41) with a CCD-camera (Colorview III, SIS, Olympus). Finally, they were measured by using the imaging software CellP (SIS). The conditions were blinded for the analysts.

### Enzyme-linked immunosorbent assay (ELISA)

The effect of HBO on the BDNF expression and release in the supernatants was investigated by performing ELISA. Thus, the BDNF concentration in the supernatants of the NIH3T3 and NIH3T3/BDNF fibroblasts was determined after the first, third and fifth HBO treatment (1 x HBO, 3 x HBO, 5 x HBO) with 2.0 bar using a human BDNF-ELISA kit (Boster biological technology Co. Ltd, Fremont, USA). Normoxic treated cells of both fibroblast cell variants at corresponding time points served as cell control. Untreated medium was used as negative control in order to exclude putative BDNF contents due to FCS within the used media. The three supernatants of the same condition and experiment were pooled.

The BDNF-ELISA kit was used following the manufacturer’s guidelines. Briefly, the standard and the samples were diluted with the provided sample dilution buffer, mixed gently and 100 μL of each diluted sample/standard was added to a well of the pre-coated 96-well plate. After an incubation period of 90 min, the sample and standard solutions were discarded. Without washing, the diluted biotinylated anti-BDNF antibody was added followed by incubation for 60 min. After washing with 0.01 M PBS (8.5 g NaCl, 1.4 g Na_2_HPO_4_, 0.2 g NaH_2_PO_4_ ad. 1 L dest. H_2_O; pH = 7.2–7.6), the wells were incubated with the provided ABC solution for 30 min. Subsequently, the plate was washed five times. The enzymatic substrate solution (3,3’,5,5’-tetramethylbenzidine, TMB) was added and the colour change was stopped with stop solution after 20 min. All incubation steps were performed at 37°C. The absorbance was measured at 450 nm using a Multiskan Ascent plate reader (Thermo Scientific Inc., Waltham, USA). Sample dilution buffer (standard curve zero value) served as blank and for analysis, all measured values were blank corrected. For statistical analysis of the BDNF concentration, ELISA was performed on supernatants collected from each condition of three independent cell culture experiments.

### Secretome analysis

For analysing the factors secreted by MSCs, MSCs were grown in cell culture flasks (75 cm^2^, TPP, Trasadingen, Schweiz). These were treated with HBO at a pressure of 2.0 bar and under normoxic conditions for five consecutive days. In beforehand of the fifth treatment, medium was exchanged and the MSCs were grown in serum-free medium in order to minimize the background of soluble factors for the secretome analysis. The secretome analysis was performed for each donor.

Acetone precipitation of proteins was made for both samples (HBO and NOR). One volume of each sample was added to five volumes of acetone and was incubated at -20°C over night. The precipitated proteins were pelleted by centrifugation at 15.000 x g at 4°C for 15 min. The pellets were washed with cold 80% acetone and dried. Proteins were solved in Laemmli buffer and incubated for 5 min at 95°C. Proteins were alkylated by adding 2 μL of an acrylamide solution (40%, w/v) and were separated by SDS-PAGE (12%). After electrophoresis, proteins were stained with Coomassie Brilliant Blue for 15 min and background staining was reduced with water. Each lane was cut into three pieces of similar size, which were further minced to 1 mm^3^ gel pieces. Sample processing was done as described [41]. Briefly, gel pieces were destained two times with 50mM NH_4_HCO_3_ in 50% acetonitrile (ACN) and with 100% ACN. The gel pieces were dried in a vacuum centrifuge and 100 μL of 10 ng/μL sequencing grade trypsin (Promega, Madison, USA) in 40 mM NH_4_HCO_3_, 10% ACN were added. Gels were rehydrated in trypsin solution for 1 hour, on ice and then covered with 40 mM NH_4_HCO_3_ in 10% ACN. Digestion was performed over night at 37°C and was stopped by adding 100 μL of 50% ACN in 0.1% trifluoroacetic acid (TFA). After incubation at 37°C for 1 hour, the solution was transferred into a fresh sample vial. This step was repeated twice and extracts were dried in a vacuum centrifuge. Dried peptide extracts were redissolved in 2% ACN in 0.1% TFA with shaking at 800 rpm for 20 min. After centrifugation at 20.000 x g, peptide samples were directly analysed with liquid chromatography–mass spectrometry (LC-MS) or stored at -20°C.

Peptide samples were separated with a nano-flow ultra-high pressure liquid chromatography system (RSLC, Thermo Scientific) equipped with a trapping column (3 μm C18 particle, 2 cm length, 75 μm ID, Acclaim PepMap, Thermo Scientific) and a 50 cm long separation column (2 μm C18 particle, 75 μm ID, Acclaim PepMap, Thermo Scientific). Peptide mixtures were injected, enriched and desalted on the trapping column at a flow rate of 6 μL/min with 0.1% TFA for 5 min. The trapping column was switched online with the separating column and peptides were eluted with a multi-step binary gradient: linear gradient of buffer B (80% ACN in 0.1% formic acid) in buffer A (0.1% formic acid) from 4% to 25% in 115 min, 25% to 50% in 25 min, 50% to 90% in 5 min and 10 min at 90% B. The column was reconditioned to 4% of buffer B in 30 min. The flow rate was 250 nL/min and the column temperature was set to 45°C. The RSLC system was coupled online via a Nano Spray Flex Ion Soure II (Thermo Scientific) to an LTQ-Orbitrap Velos mass spectrometer. Metal-coated fused-silica emitters (SilicaTip, 10 μm i.d., New Objectives) and a voltage of 1.3 kV were used for the electrospray. Overview scans were acquired at a resolution of 60 k in a mass range of m/z 300–1600 in the orbitrap analyzer and stored in profile mode. The top 10 most intensive ions of charges two or three and a minimum intensity of 2000 counts were selected for CID fragmentation with a normalized collision energy of 38.0, an activation time of 10 ms and an activation Q of 0.250 in the LTQ. Fragment ion mass spectra were recorded in the LTQ at normal scan rate and stored as centroid m/z value and intensity pairs. Active exclusion was activated so that ions fragmented once were excluded from further fragmentation for 70 s within a mass window of 10 ppm of the specific m/z value. Raw data were processed using Max Quant software (version 1.4) and human entries of Uniprot database. Threshold for protein identification was set to 0.01 on peptide and protein level Analyses of protein abundances and calculations of up or down regulated proteins were done with the Perseus software (version 1.5.30).

### Statistical analysis

Statistical analyses were performed with Prism 5 (GraphPad, La Jolla, USA). The results were validated by using one-way ANOVA followed by Bonferroni’s multiple comparison test. P values of less than 0.05 were considered to be statistically significant. All data represent the means of three independent approaches (N), including triplicates of each sample (n) (N = 3, n = 3), except the secretome analysis (one analysis per donor) and the ELISA experiments (N = 2, n = 3). Error bars in the figures indicate the standard error of the mean (SEM). Levels of significance are indicated as follows: *p < 0.05; **p < 0.01; ***p < 0.001.

## Results

### Effects of HBO on proliferation of fibroblasts and MSCs

**Fig 1:**
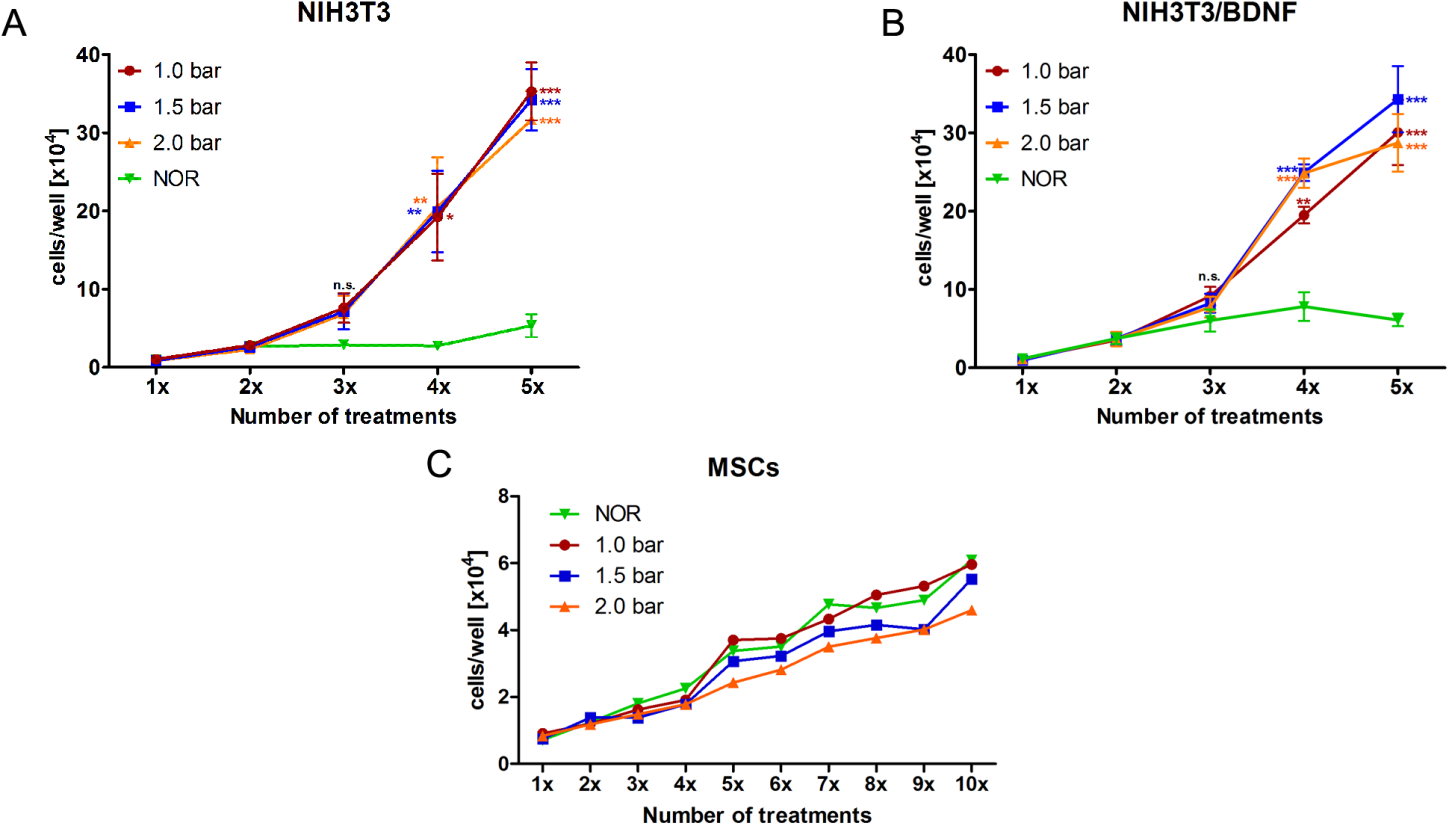
Proliferation of HBO-treated cells. The effect of hyperbaric oxygen (HBO) treatment on the proliferation of NIH3T3 fibroblasts (A), genetically modified NIH3T3/BDNF fibroblasts (B) and MSCs (C) is depicted. HBO was applied at three different pressures: 1.0 bar (red circle and line), 1.5 bar (blue square and line) and 2.0 bar (orange triangle and line). Both NIH3T3 and NIH3T3/BDNF fibroblasts show a highly significantly increased cell number compared to the control cells (normoxic and normobaric conditions (NOR), green inverted triangle and line) after five consecutive HBO treatments. By contrast, the proliferation of MSCs started to decrease after five consecutive HBO treatments with 2.0 bar until the end of ten treatments in comparison to NOR. Values are given as mean ± standard error of the mean (SEM); N = 3, n = 3. Asterisks indicate the significance of cell numbers of the different used pressures compared to the normoxic control. Statistical assessment was performed using one-way ANOVA with Bonferroni’s multiple comparison test (n.s. = not significant; *p < 0.05; **p < 0.01; ***p < 0.001).

After plating of fibroblasts, cell proliferation and cell numbers increased from day to day in each condition regardless of the specific treatment (Fig 1A and 1B). From the third treatment on, differences between HBO-treated cells and cells grown under normoxic conditions (NOR) were observed for both fibroblast cell types (native cells, BDNF-expressing cells): the proliferation of NIH3T3/BDNF and NIH3T3 fibroblasts started to increase significantly under the influence of HBO whereas control fibroblasts, especially NIH3T3, showed only a minimally increased cell number. With the fourth HBO treatment, the proliferation of NIH3T3 fibroblasts was significantly increased (1 bar: 19.21 ± 5.53 x 10^4^ cells/well, p < 0.05; 1.5 bar: 19.92 ± 5.20 x 10^4^ cells/well and 2.0 bar: 20.49 ± 6.36 x 10^4^ cells/well, p < 0.01) when compared to the normoxic control (NOR: 2.77 ± 0.27 x 10^4^ cells/well) (Fig 1A). This increase in proliferation was also observed for NIH3T3/BDNF fibroblasts (1 bar: 19.49 ± 1.04 x 10^4^ cells/well, p < 0.01; 1.5 bar: 24.92 ± 1.07 x 10^4^ cells/well and 2.0 bar: 24.84 ± 1.88 x 10^4^ cells/well, p < 0.001) compared to their control (NOR: 7.82 ± 1.83 x 10^4^ cells/well) (Fig 1B). After five HBO treatments, NIH3T3/BDNF fibroblasts exposed to HBO at a pressure of 1.5 bar exhibited the highest cell numbers (34.29 ± 4.22 x 10^4^ cells/well) and this increase was highly significant when compared to the normoxic control (Fig 1B). However, the treatment with HBO at 1.0 bar and 2.0 bar for five times also led to a highly significant increase of the cell numbers of both types of fibroblasts when compared to the normoxic control.

**Fig 2:**
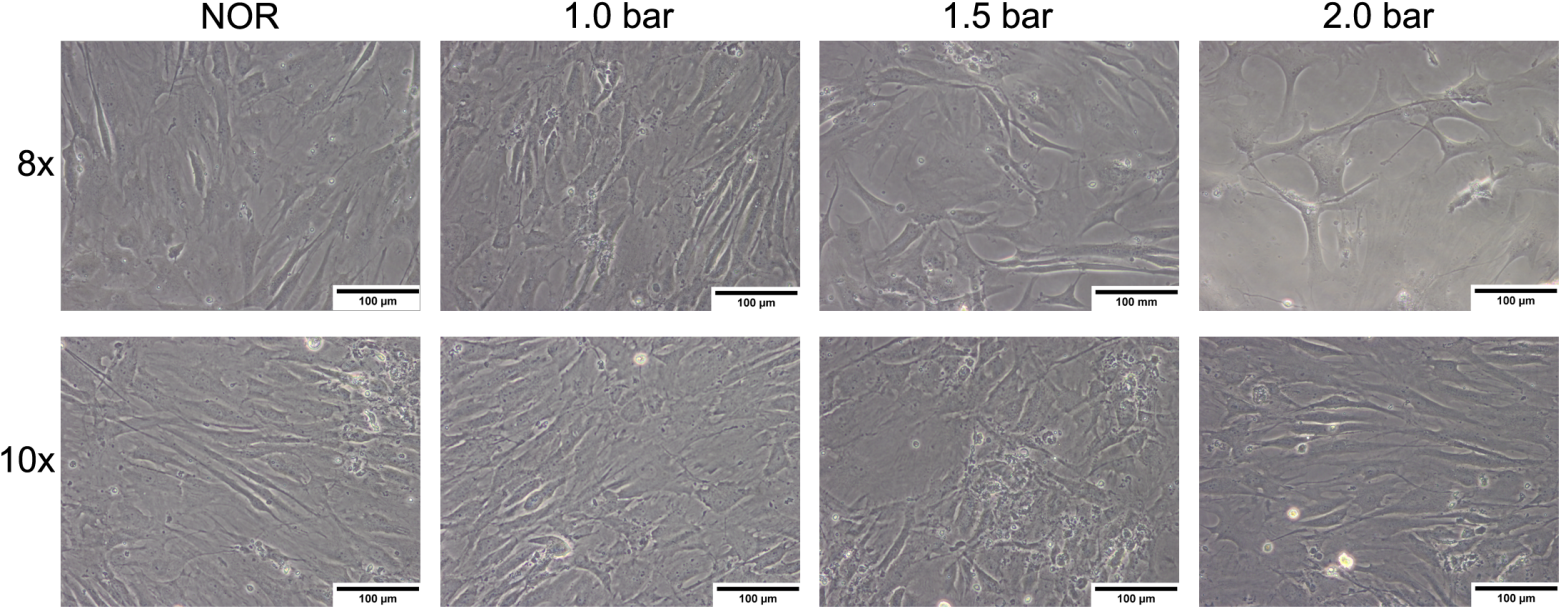
Representative images of HBO-treated MSCs. Microscopic brightfield images demonstrate the effect of HBO treatment on MSCs. Decreased cell numbers were observed after five (not shown), eight and ten treatments (corresponding to eight, 11 and 15 days of *in vitro* cultivation, respectively) in MSCs treated with HBO at 2.0 bar. By contrast, HBO treatment with 1.0 bar showed a cell density comparable with the control (NOR). All pictures are presented at a 200-fold magnification. Scale bar: 100 μm.

The cell number of HBO-treated MSCs increased from day to day until the fourth treatment (Fig 1C). From the fifth HBO treatment onwards, for MSCs treated with 2.0 bar of HBO a decreased cell number (2.43 ± 0.44 x 10^4^ cells/well, p = n.s.) was observed, when compared to other conditions (NOR: 3.38 ± 0.40 x 10^4^ cells/well; 1.0 bar: 3.71 ± 0.44 cells/well and 1.5 bar: 3.07 ± 0.47 cells/well). This effect remained until the end of the tenth treatment. The microscopic images of the MSCs corroborated the observed results concerning the cell density (Fig 2) especially when the normoxic treatment is compared with the MSCs treated with 2.0 bar of HBO for eight times (Fig 2, first row).

### Effect of HBO on BDNF-secretion of tested cells

In order to investigate the endogenous BDNF release of oxygen-treated cells, ELISA was performed for the quantification of the BDNF level in the conditioned medium. MSCs did not secrete a detectable amount of BDNF (0.01 ± 0.01 ng/mL; data not shown). Supernatants from NIH3T3 fibroblasts contained only a mean BDNF amount of 0.07 ± 0.01 ng/mL which is comparable to the medium control (0.0 ng/mL; data not shown).

**Fig 3:**
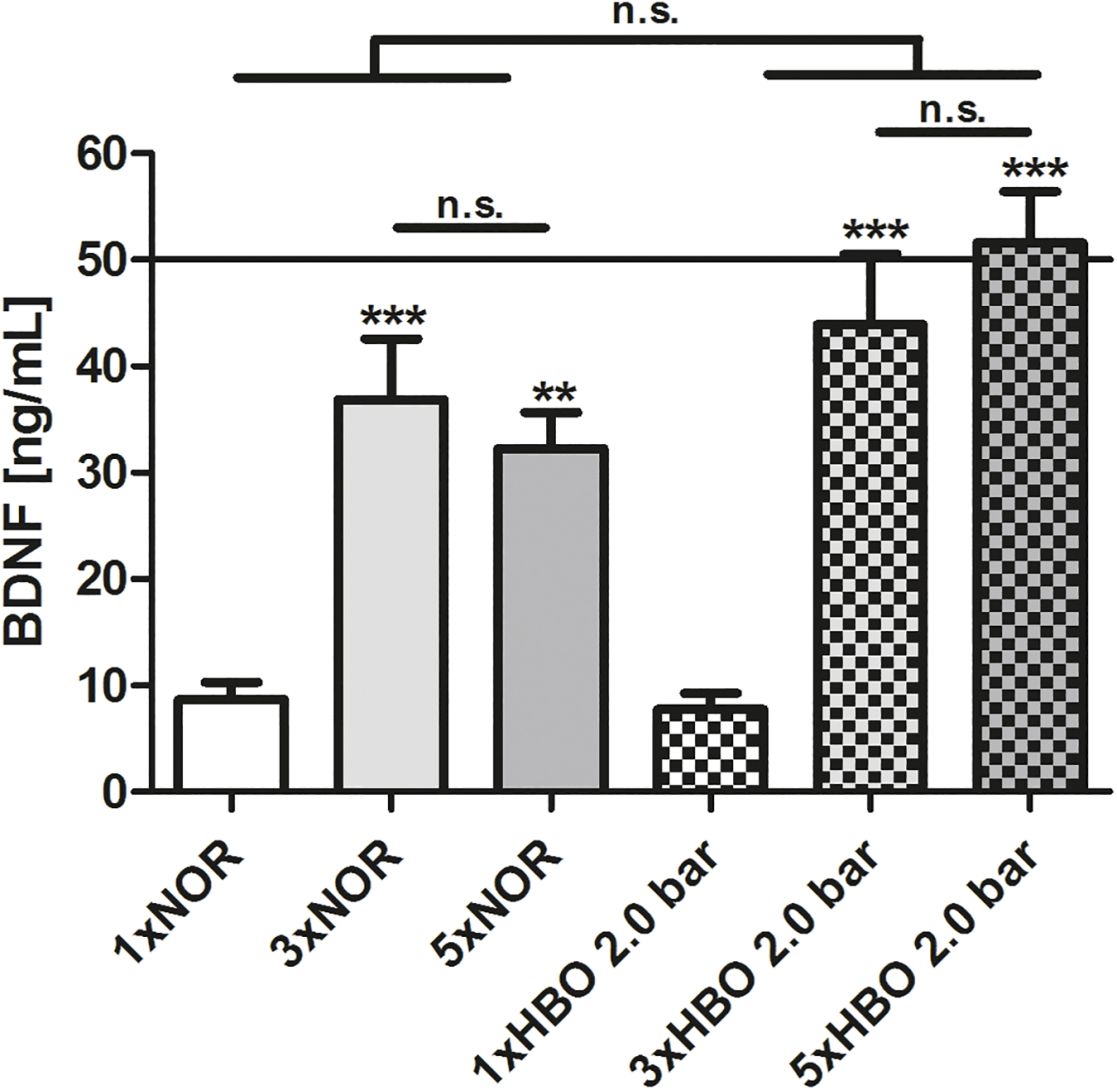
Effect of HBO treatment on BDNF-secretion of genetically modified fibroblasts. Quantitative determination of BDNF secreted by recombinant NIH3T3/BDNF fibroblasts exposed to HBO at an oxygen pressure of 2.0 bar (intensely dotted) and their corresponding controls (NOR, without pattern) by ELISA. The release of BDNF is significantly increased after three and five consecutive HBO treatments and three to five NOR treatments (light grey and grey) when compared to their respective controls. The continuous line represents the recommended BDNF concentration (50 ng/mL) for optimal neuronal survival of SGN. All values are given as mean ± standard error of the mean (SEM). Statistical analysis was performed by one-way ANOVA with Bonferroni’s correction (n.s. = not significant; *p < 0.05; **p < 0.01; ***p < 0.001), comparing the respective single treatment value (1xNOR, 1xHBO) to the repeated treatment.

For a representative analysis of the secreted BDNF in the supernatants from NIH3T3/BDNF fibroblasts, the conditioned medium from fibroblasts treated with HBO of 2.0 bar was selected (Fig 3). A single HBO treatment resulted in a mean concentration of 7.80 ± 1.48 ng/mL BDNF. The BDNF concentration increased in a highly significant manner after three consecutive HBO treatments up to 43.94 ± 6.59 ng/mL (p < 0.001) and after five HBO treatments up to 51.63 ± 4.80 ng/mL (p < 0.001) when compared to the BDNF concentration after one HBO treatment. The BDNF amount after five HBO treatments is comparable to the optimal concentration of BDNF (50 ng/mL, black horizontal line) [40], which was used as the BDNF control in the SGN experiments. Under the normoxic treatment of NIH3T3/BDNF fibroblasts at ambient pressure (NOR), secreted BDNF also significantly increased from 8.67 ± 1.65 ng/mL (1xNOR) to 36.88 ± 5.71 ng/mL (3xNOR) and 32.27 ± 3.42 ng/mL (5xNOR). However, no significant differences in the amount of secreted BDNF were found between the HBO-treated and the normoxic and normobaric groups after comparable cycles of treatments. Relating the measured mean BDNF concentration to the corresponding mean of cell number resulted in a BDNF-release of 0.62 pg/cell after one treatment for HBO-treated cells and 0.64 pg/cell for NOR-treated cells (S1 Fig). After five treatments, the BDNF secretion per cell decreased to 0.52 pg/cell for the normoxic control and to 0.20 pg/cell for the HBO-treated fibroblasts.

### Survival rate of SGN after cultivation in supernatants of HBO-treated cells

**Fig 4:**
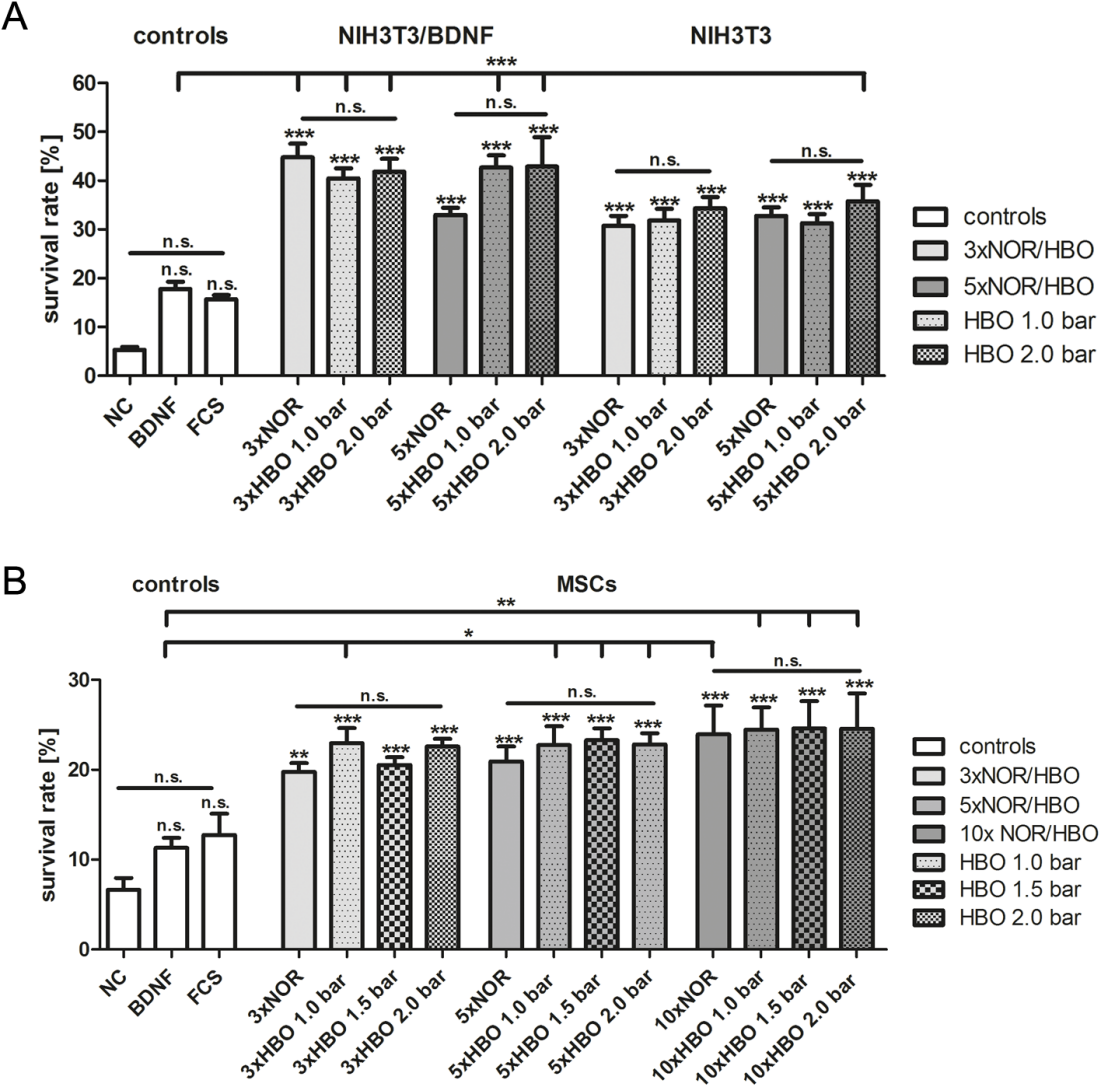
Survival rates of SGN cultivated in supernatants obtained from HBO-treated cells. Mean survival rates of spiral ganglion neurons (SGN) were determined by the amount of neurons in relation to the seeding control after cultivation in supernatants obtained from HBO-treated NIH3T3 and NIH3T3/BDNF fibroblasts (A) and MSCs (B). The treatment with supernatants significantly enhanced the survival of SGN compared to the negative control (NC, white column without pattern). Values are given as mean ± standard error of the mean (SEM); N = 3, n = 3; Controls: NC (spiral ganglion medium control, serum-free), BDNF (spiral ganglion medium substituted with 50 ng/mL human recombinant BDNF, positive control), FCS (fetal calf serum containing fibroblast/MSC medium, medium control). Statistical analysis was performed using one-way ANOVA with Bonferroni’s multiple comparison test (n.s. = not significant; *p < 0.05; **p < 0.01; ***p < 0.001). Asterisks over the error bars of the columns indicate the significance compared to the NC.

To investigate the bioactivity and the neuroprotective effect of oxygen-treated (NOR and HBO) NIH3T3 fibroblasts, NIH3T3/BDNF fibroblasts and MSCs, SGN were cultivated in collected supernatants. The survival rate of SGN after incubation in these supernatants is depicted in Fig 4. A negative control (NC; SGN in serum-deprived SGN medium), a positive control (BDNF; SGN in SGN medium supplemented with 50 ng/mL BDNF) and a FCS-containing medium control (white columns) were included. Irrespective of the oxygen treatment (NOR or HBO), a highly significantly increased survival rate was obtained after cultivation of SGN in supernatants from NIH3T3 (ranging from 30.73 ± 2.05 to 35.77 ± 3.35%) and NIH3T3/BDNF fibroblasts (ranging from 32.97 ± 1.44 to 44.78 ± 2.81%) compared to the negative control (5.33 ± 0.60%) (Fig 4A). Also compared to the negative control, survival was increased after the cultivation of SGN in medium supplemented with BDNF (17.77 ± 1.48%) and FCS (15.66 ± 0.86%) (Fig 4A). Although supernatants of HBO-treated NIH3T3/BDNF fibroblasts accounted for higher survival rates in SGN when compared to NIH3T3 fibroblasts, the difference was not statistically significant. After five consecutive oxygen treatments, a tendency towards an increased survival of SGN treated with 2.0 bar of HBO was observed. Three treatments led to a highly significant increase of the survival rate for supernatants of NOR- and HBO-treated NIH3T3/BDNF fibroblasts whereas after five treatments only supernatants of HBO-treated NIH3T3/BDNF fibroblasts (i.e., 1.0 bar and 2.0 bar) resulted in highly significantly increased survival rates when compared to the BDNF control, respectively. From NIH3T3 fibroblasts only supernatants of five times HBO-treated cells showed a highly significantly increased survival rate in comparison to the BDNF control.

The survival rate of SGN cultivated in conditioned medium from MSCs (ranging from 19.77 ± 0.96% to 24.60 ± 3.05%), irrespective of the treatment (NOR or HBO), was highly significantly increased when compared to the negative control (6.65 ± 1.31%) (Fig 4B). SGN cultivated in medium supplemented with 50 ng/mL of human recombinant BDNF (11.31 ± 1.10%) and in MSC medium which contained FCS (12.73 ± 2.39%) resulted in an increased, but not significant, survival rate when compared to the NC (Fig 4B, white columns without pattern). The highest survival rates were obtained after 10 times of NOR and HBO treatment (ranging from 23.94 ± 3.20% to 24.60 ± 3.05%). After five treatments, only supernatants of HBO-treated MSCs showed a significantly increased survival rate when compared to the BDNF control whereas after ten treatments all tested conditions were significantly increased.

### Neurite length of SGN after cultivation in supernatants of HBO-treated cells

**Fig 5:**
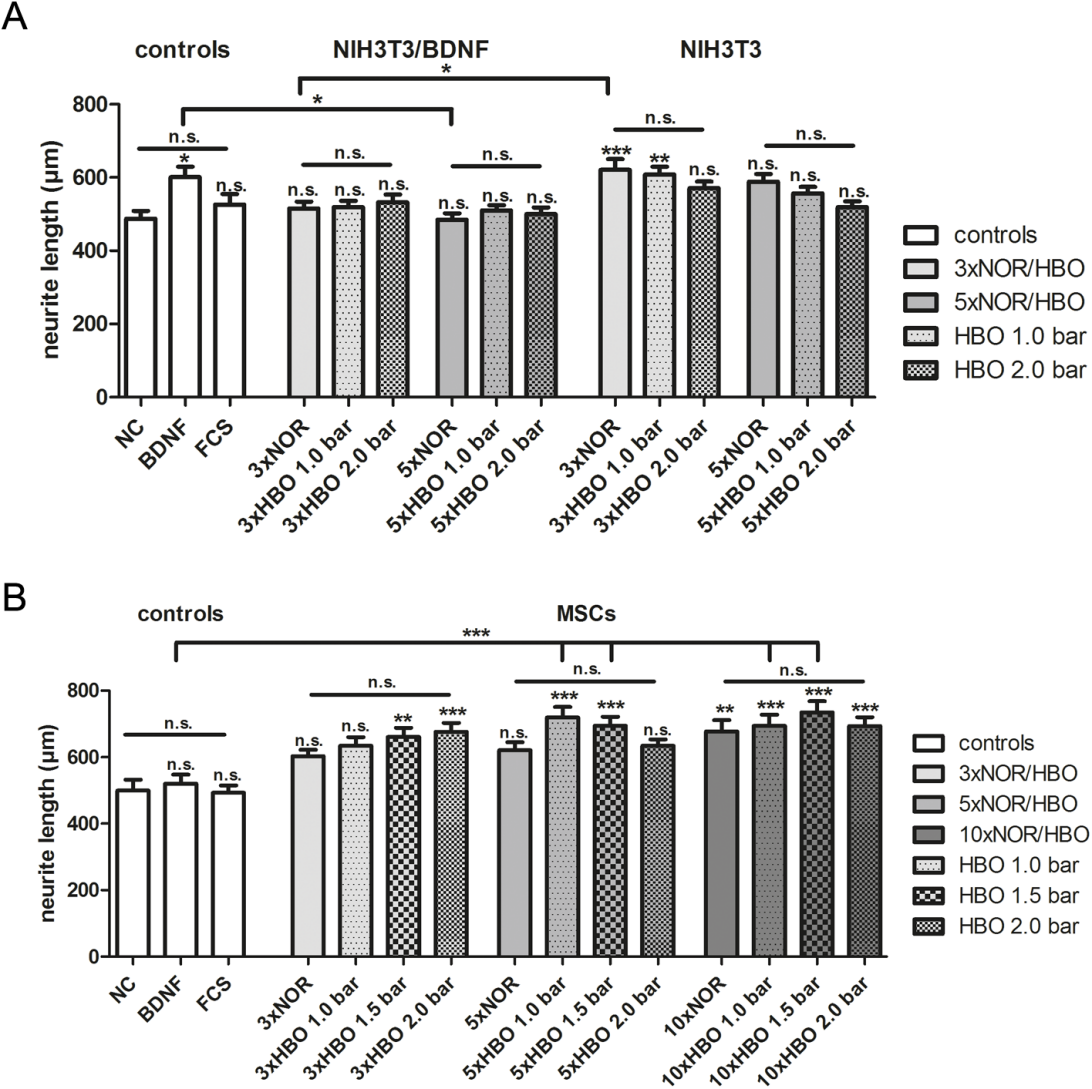
Neurite length of SGN cultivated in supernatants obtained from HBO-treated cells. Mean neurite length of SGN, treated with supernatants from HBO-treated and NOR-treated NIH3T3 as well as NIH3T3/BDNF fibroblasts (A) and MSCs (B), are shown. Supernatants of 1.0 and 1.5 bar HBO-treated MSCs significantly increased the neurite length in comparison to the BDNF control and negative control. The asterisks over the error bars of the columns refer to the NC. N = 3; n = 3; n.s. = not significant; *p < 0.05; ** p< 0.01; ***p < 0.001.

For the evaluation of the neuroregenerative effect of HBO on SGN, the neurite length of the surviving SGN was measured and the mean neurite length is depicted in Fig 5. Within the control groups in the fibroblast experiments (Fig 5A), the longest neurites were detected in the BDNF control (BDNF; 601.3 ± 28.55 μm) and were significantly increased when compared to the negative control (NC: 487.8 ± 21.34 μm). The overall longest neurites were measured in SGN cultures exposed to supernatants of NIH3T3 fibroblasts treated three times with 1.0 bar oxygen (3xHBO 1.0 bar; 608.1 ± 21.22 μm) and their corresponding normoxic control (3xNOR: 620.8 ± 29.32 μm). This neurite outgrowth of the latter one was the only detected statistically highly significant increase in neurite length when compared to the NC. Interestingly, cultivation with supernatants of NIH3T3 fibroblasts (ranging from 519.2 ± 16.14 μm to 620.8 ± 29.32 μm) resulted in longer neurites than supernatants of NIH3T3/BDNF (ranging from 485.1 ± 16.97 μm to 532.5 ± 21.00 μm). Moreover, an increased pressure of HBO resulted in a reduced neurite length of SGN (from 608.1 ± 21.22 μm in 1.0 bar to 571.3 ± 17.63 μm in 2.0 bar) only in SGN grown in the supernatants of NIH3T3 fibroblasts.

The cultivation of SGN in supernatants collected from HBO- and NOR-treated MSCs led to an increased neurite length (ranging from 603.00 ± 19.04 μm to 735.20 ± 32.87 μm) in comparison to the negative control (499.60 ± 32.53 μm). After three treatments, the neurites of SGN treated with supernatants of HBO-treated MSCs (1.5 bar and 2.0 bar) were significantly longer than in the negative control. Five HBO treatments of MSCs at 1.0 bar and 1.5 bar resulted in significantly longer neurites than in the negative control whereas ten treatments gave rise to significantly longer neurites in all conditions in comparison to the negative control. Even supernatants of five and ten times HBO-treated MSC at 1.0 bar and 1.5 bar significantly increased the neurite length of survived SGN in comparison to the BDNF-control.

### Differences in the secretome of HBO-treated MSCs compared to normoxic control

For analyzing the effects of different percentages of oxygen (22% and 100%) and pressures (1.0 bar and 2.0 bar) on human MSC secretome, a secretome analysis was performed. The results showed that different oxygen percentages and pressures modulated the secretome of MSCs to produce different expression patterns, in which the HBO treatment (100%, 2.0 bar) led to a slightly increased secretion profile of proteins in MSCs when compared to the normoxic control. In total, it was possible to identify 282 proteins, whereof 16 proteins were identified only in the secretome of HBO-treated MSCs and one protein only in MSCs treated with normoxic conditions. Therefore, the two tested conditions have 265 proteins in common from which 37 were upregulated (Table 2) in HBO-treated MSCs when compared to the normoxic control condition and 22 were downregulated (Table 3). Some of the detected upregulated proteins are involved in cellular mechanisms against oxygen stress, e.g. Thioredoxin, Heat shock protein (HSP) 90, HSP70, Gamma-glutamylcyclotransferase, Thrombospondin-4 and Vasorin. Regarding neuroregulatory potential, Peroxiredoxin, Cystatin C, Laminin, Syndecan and Thymosin-beta expression were found in the common secreted proteins.

**Table 2:** Proteins upregulated in HBO-treated MSCs in comparison to NOR-treated MSCs.

Protein name	Protein ID	Mean regulation
Keratin, type I cytoskeletal 19	K7EMS3	-3,3265
Ubiquitin carboxyl-terminal hydrolase isozyme L3	Q5TBK7	-3,31831
Coiled-coil domain-containing protein 170	Q8IYT3	-2,43056
Calmodulin-like protein 5	Q9NZT1	-2,35419
Tubulin-specific chaperone D	J3KR97	-2,35055
Glia-derived nexin	P07093	-2,3191
Heat shock protein HSP 90-beta;	P08238;	-1,92572
Heat shock protein HSP 90-alpha	P07900	
GTP-binding nuclear protein Ran	B5MDF5	-1,85749
Swi5-dependent recombination DNA repair protein 1 homolog	Q86XK3	-1,83976
Suprabasin	Q6UWP8	-1,83319
Myeloid-derived growth factor	Q969H8	-1,77311
Thioredoxin	P10599-2	-1,76304
Microprocessor complex subunit DGCR8	Q8WYQ5-3	-1,67307
ATP synthase subunit alpha, mitochondrial	P25705-2	-1,62953
Recombining binding protein suppressor of hairless-like protein	Q5QPV1	-1,62211
Fatty acid-binding protein, epidermal	Q01469	-1,60576
Serpin B12	Q96P63	-1,59943
Cystatin-A	P01040	-1,50903
Aggrecan core protein	H0YM81	-1,50155
Transaldolase	F2Z393	-1,48908
Single-stranded DNA-binding protein	C9K0U8	-1,46483
Galectin-7	P47929	-1,41972
Gamma-glutamylcyclotransferase	H7BZK5	-1,39692
60 kDa heat shock protein	P10809	-1,38681
Thrombospondin-4	E7ES19	-1,35424
Dermcidin	P81605	-1,32876
Malate dehydrogenase	G3XAL0	-1,27935
Olfactomedin-like protein 2B	Q68BL8	-1,23622
Peptidyl-prolyl cis-trans isomerase FKBP7	Q9Y680	-1,22746
Zinc-alpha-2-glycoprotein	A8MT79	-1,2165
Lactotransferrin	E7EQB2	-1,20696
Neuroblast differentiation-associated protein AHNAK	Q09666	-1,20539
Vasorin	Q6EMK4	-1,20532
U1 small nuclear ribonucleoprotein A	P09012	-1,15938
60S ribosomal protein L29	P47914	-1,11455
Dynein light chain roadblock-type 1;	H3BNG9;	-1,02755
Dynein light chain roadblock-type 2	H3BPA0	
Protein-glutamine gamma-glutamyltransferase K	P22735	-1,02732

The data were generally transformed to log scale (log2); - 1 = fold change of 2; - 2 = fold change 4; -3 = fold change 8.

**Table 3:** Proteins downregulated in HBO-treated MSCs in comparison to NOR-treated MSCs.

Protein name	Protein ID	Mean regulation
**SH3 domain-binding glutamic acid-rich-like protein 3**	SH3BGRL3	2,62589
**Phosphoglycerate mutase 1;**	PGAM1;	2,55078
**Phosphoglycerate mutase 2;**	PGAM2;	
**Phosphoglycerate mutase 4**	PGAM4	
**ATP synthase subunit beta**	ATP5B	2,19197
**Anthrax toxin receptor 1**	ANTXR1	1,90491
**Exocyst complex component 3-like protein 4**	EXOC3L4	1,75485
**Ferritin**	FTH1	1,72806
**Zinc finger protein 271**	ZNF271	1,65265
**Glypican-1;Secreted glypican-1**	GPC1	1,53348
**Histone H2A**	H2AFV	1,49156
**Out at first protein homolog**	OAF	1,40593
**Prelamin-A/C; Lamin-A/C**	LMNA	1,34475
**Phosphate carrier protein, mitochondrial**	SLC25A3	1,32169
**72 kDa type IV collagenase; PEX**	MMP2	1,31056
**Calumenin**	CALU	1,29926
**Sushi repeat-containing protein SRPX2**	SRPX2	1,14635
**Syndecan**	SDC2	1,13812
**Serpin H1**	SERPINH1	1,11928
**Lumican**	LUM	1,07974
**Minor histocompatibility protein HB-1**	HMHB1	1,0612
**Target of Nesh-SH3**	ABI3BP	1,04938
**Proteasome subunit alpha type-5**	PSMA5	1,04372
**Clusterin**	CLU	1,00908

The data were generally transformed to log scale (log2); + 1 = fold change of 2; + 2 = fold change 4.

## Discussion

The current study demonstrated that HBO treatment notably enhanced the proliferation of NIH3T3 and genetically modified NIH3T3/BDNF fibroblasts, in contrast to human MSCs with non-altered or even slightly reduced proliferation at 2.0 bar. A halted or reduced proliferation of MSC is important when considering cell transplantation to the cochlea in order to prevent excessive growth of cells and obliteration of the cochlea. Nevertheless, HBO treatment promoted an enhanced survival of SGNs when treated with supernatants of both fibroblasts and MSCs. In the MSC culture, HBO treatment resulted in an additional production of proteins with neuroprotective potential.

Our results revealed a 36-fold increase in cell number for the HBO-treated NIH3T3 fibroblasts and a 29-fold increase in cell number for HBO-treated NIH3T3/BDNF fibroblasts each compared to the first HBO treatment. This was in contrast to Dimitrijevich et al. (1999) [42], who detected only a ten-fold increase of the cell numbers in human skin equivalents after ten days of HBO treatment and to Conconi et al. (2003) [43], who found a six-fold increased cell number in 3T3/J2 fibroblasts after HBO treatment in a mouse fibroblast cell line. The normoxic controls in our study confirmed the findings of Dimitrijevich and colleagues (1999) [42] by revealing a five-fold increase in cell number. The proliferation of human MSCs was affected in the opposite way: From the fifth HBO treatment on, the MSCs treated with 2.0 bar of HBO started to proliferate slower compared to the control MSCs and MSCs treated with lower pressures of HBO. The normoxic control revealed a nine-fold increased cell number after ten HBO treatments, whereas the HBO-treated MSCs showed an eight-fold (1.5 bar), a seven-fold (1.0 bar) and a six-fold (2.0 bar) increased cell number in comparison to the first treatment. Focussing on the cell numbers after the tenth HBO treatment revealed that the cell number of MSCs treated with 2.0 bar of HBO is decreased by 25% when compared to the other three conditions. Pure oxygen (at a pressure of 1.0 bar) did not influence the proliferative behavior of MSCs because the HBO treatment with 1.0 bar shows no differences to the normoxic control, whereas at a pressure of 1.5 bar small negative influences on the cell numbers were observed. Usually, MSCs exist in perivascular niches, which exhibit low oxygen pressure [26]. The oxygen level in human bone marrow, from which the MSCs were isolated, is between 1% and 7% [27]. Possibly, maintenance of MSCs in an undifferentiated state may require a hypoxic environment and high oxygen tensions may promote differentiation and the MSCs left the cell cycle which reduces the proliferation. In the fibroblast experiments we demonstrated that even pure oxygen at normobaric pressure (1.0 bar) significantly increased the proliferation of both types of NIH3T3 fibroblasts in comparison to the normoxic control.

Changes in the pH of cultivation medium during HBO treatment could be neglected because Dimitrijevic et al. (1999) [42] reported that during a 90-minute treatment the changes in the pH were less than 0.1 pH units and not significant. Besides, the used culture medium has a sodium bicarbonate buffer system with phenol red as a pH indicator. However, colour shifts indicating pH changes were not observed throughout the experiments with one exception: after 7 days (5 x HBO) in the fibroblast experiments, the cells were confluent and the high cell numbers caused a slight pH shift.

The increased survival rates of SGN after the treatment with supernatants of HBO-treated NIH3T3/BDNF fibroblasts correlated with the concentration of secreted BDNF as determined by ELISA. Our results revealed a five-fold increased BDNF-release from NIH3T3/BDNF fibroblasts after five HBO treatments compared to one HBO treatment, but the normoxic control showed also a four-fold increase of secreted BDNF. Therefore, the five times HBO treated NIH3T3/BDNF showed the highest amount of BDNF with a BDNF concentration of 51.63 ± 4.80 ng/mL (5xHBO) which is very close to the recommended BDNF concentration of 50 ng/mL [40] for an enhanced SGN survival. But this increase is not as high as expected due to the highly significantly increased cell numbers of the HBO-treated fibroblasts. Relating the mean BDNF concentration to the mean cell number showed an obviously decreased BDNF secretion per cell for the HBO-treated fibroblasts which is obviously due to confluence accompanied by shortage of nutrients. The supernatants of both types of HBO-treated NIH3T3 fibroblasts increased the survival of SGN in comparison to the negative control (serum deprived) and demonstrated that HBO treatment did not interfere with the neuroprotective effect and that already FCS in combination with secreted factors of cells enhanced the survival of SGN. Additionally, the survival rates of SGN treated with the supernatants of HBO-treated NIH3T3/BDNF fibroblasts were very close to the survival rate of the factor combination of FCS and BDNF (50 ng/mL, data not shown), which corroborated the bioactivity of the secreted BDNF in combination with the FCS of the fibroblast medium. Supernatants of five times NOR-treated NIH3T3/BDNF fibroblasts resulted in a lower survival rate, which was comparable to the survival rate of SGN treated with supernatants of NIH3T3 fibroblasts. This is quite interesting with regard to the measured BDNF concentration of 32.27 ng/mL for five times NOR-treated NIH3T3/BDNF fibroblasts and about 0.07 ng/mL for the NIH3T3 fibroblasts. This could be a hint, that HBO has the ability to modulate (enhance) survival but this seems not be caused by enhancement or upregulation of intracellular BDNF factor production, it might be through modulation of the downstream signalling. The survival rates of SGN, which were treated with supernatants of HBO- and NOR-treated MSCs were also significantly increased in comparison to the negative control (serum deprived). But only supernatants of three and five times HBO-treated MSCs increased the survival rate of SGN in comparison to the BDNF control, indicating that the oxygen treatment slightly improves the neuroprotective properties.

Supernatants contain a lot of other cell secreted proteins and growth factors, which could be growth-enhancing or -inhibiting. The presence of growth-enhancing factors other than BDNF is very likely due to the fact that supernatants of HBO-treated NIH3T3 fibroblasts exerted neuroprotective actions on SGN when compared to the BDNF and FCS control (Fig 4; NIH3T3 vs. FCS/BDNF). This was corroborated by the fact that BDNF was not detected by ELISA in NIH3T3 (data not shown). Since the expression of other neurotrophic factors like ciliary neurotrophic factor, nerve growth factor or NT-3 has not been reported for NIH3T3 fibroblasts, we assume that the neuroprotective and neuroregenerative effects of NIH3T3 fibroblasts may result from FCS in combination with other secreted factors or from bystander cells (glial cells), which were present in the primary SGN culture. The glial cells are known to release neurotrophic factors, which support the protection of SGN and the induction of neurite outgrowth. Schwieger et al. (2015) [44] showed that the neuronal survival and neurite outgrowth were significantly increased with the combination of BDNF and ciliary neurotrophic factor compared to the treatment with each of these factors alone. Moreover, only a small amount of BDNF (5 ng/mL) is required for the induction of neuritogenesis [14], but the survival of neurons needed a higher and nearly constant BDNF level, ranging from 25 ng/mL to 100 ng/mL with highest survival rates at a BDNF concentration of 50 ng/mL [40]. The results of the present study confirmed that cell-derived human BDNF has a neuroprotective effect on SGN. Several other studies showed an enhanced SGN survival *in vitro* [13,37,40,45] and *in vivo* [15,46,47], which was mediated by BDNF. However, an analogous neuritogenesis as determined by measuring the neurite length of SGN was not observed in the herein presented study. This finding is corroborated by Jin et al. (2013) [48] showing a rather inhibited neurite extension in SGN cultures (postnatal day 5) after the treatment with BDNF and the beneficial and harmful effects of BDNF on the auditory system was debated in a recent review [49]. Additionally, maximal neuronal survival was reported for SGN after the treatment with 50 ng/mL BDNF, although the neurite outgrowth was very limited [50]. Previous studies showed that human bone marrow-derived MSCs secreted also small quantities of BDNF and other neurotrophic factors such as nerve growth factor, neurotrophin-3, glial cell line-derived neurotrophic factor and fibroblast growth factor-2 which have been described as neuroprotective factors for SGN [21,51]. Moreover, the survival rate of neurons was increased after cultivation in MSCs conditioned medium [51] and was corroborated for SGN in this study. Supernatants of MSCs were per se neuroprotective for SGN and HBO did not alter this effect.

The secretome analysis revealed that HBO-treated MSCs secrete slightly different proteins when compared to normoxic control cells. MSCs were able to secrete molecules with neuroprotective potential, e.g., Peroxiredoxin, Cystatin C, Laminin, Syndecan and Thymosin-beta expression. Peroxiredoxin, Cystatin C and Thymosin-beta have already been found by Teixeira et al. (2015) [52] in human umbilical cord Wharton Jelly-derived mesenchymal stem cells and the secretion of Thymosin-beta was significantly upregulated after cultivation under hypoxic conditions. After HBO treatment, MSCs secreted some additional proteins which were related to cellular responses to oxidative stress, e.g. Thioredoxin, Heat shock protein (HSP) 90, HSP70, Gamma-glutamylcyclotransferase, Thrombospondin-4 and Vasorin, and these proteins were upregulated when compared to the normoxic control. Additionally, some of these upregulated proteins had also neuroprotective potential, e.g. HSP70, Thromospondin-4 and Thioredoxin [6,53–56]. Although it is often reported that the oxygen concentration is an important regulator of the therapeutic and differentiation potential of MSCs, but even these differences in protein secretion due to high oxygen levels did not alter the neuroprotective effect of MSCs. However, the physiologic oxygen concentration ranged from 1% to 7% in the bone marrow and is much lower than the administered pressure during an HBO treatment. Nevertheless, neither low oxygen concentration (5%; [52]) nor high oxygen concentrations (100% daily for 90 min; current study) seemed to influence the neuroprotective behavior of MSCs, only the protein secretion profile is affected. Anyway, the obtained differences in protein secretion were not strong enough to induce significant changes in the functional properties of MSCs.

The mode of action of HBOT is complex and involves the interaction of many different events like formation of reactive oxygen species (ROS) and activation of cellular signalling pathways. Several studies reported on positive effects of HBOT, e.g., better wound healing [20], increased cell proliferation of fibroblasts [42,43,57], enhanced growth factor production [58] and improved glycosaminoglycan and collagen production [59]. By contrast, one study observed an increased production of glycosaminoglycans in cultured fibroblasts with decreased proliferation rates of 7% [60] and others mentioned toxic effects. Adverse effects of HBOT could result from the presence of high oxygen concentrations, which lead to the production of nitric oxide (NO) or ROS [61]. However, Conconi and colleagues (2004) [43] showed that there is no evident relationship between the effect of HBOT on cell proliferation and ROS production. Moreover, NO could play a modulating role in cell proliferation and growth factor expression [61] by mediating a proliferative arrest of the cell cycle, starting a differentiation programme and inducing repair-directed neurogenesis [62]. The function of NO depends on its concentration, which varies during consecutive HBO treatments [61]. NO decreased the survival of neurons but the cultivation in MSC conditioned medium reduced this effect [51]. Due to the administered high pressure, the cells could be also mechanically damaged. All these findings indicated that there is only a thin line between enough and too much oxygen and pressure concerning a positive or negative modulation of cell behaviour. High pressures, e.g., higher than 2.0 and 3.0 bar, increased cell death of human fibroblasts [42]. In addition, the effect of HBO treatment depends on the cell type, the exposure time and the amount of pressure [63]. Tompach et al. (1997) [64] proved the effect of a single HBO treatment on skin fibroblasts under a wide range of pressures and showed an increased cell proliferation at 2.4 bar. Pressures up to 4.0 bar inhibited cell proliferation. Dimitrijevich et al. (1999) [42] analysed the effect of HBO treatment with a duration of 90 minutes on three different cell types. They showed an increased cell number after ten days of treatment with 1.0 bar for human fibroblasts, whereas a treatment with 3.0 bar was not stimulating and perhaps anti-mitotic. HBO at low chamber pressure like 1.0 bar for an exposure of 60 minutes enhanced the cell growth of a fibroblast cell line (3T3/J2), whereas a longer exposure and higher pressure (2.5 bar) decreased the cell growth [43]. Kang et al. (2004) [59] reported an initial decrease in cell proliferation but a daily treatment of HBO at 2.0 bar stimulated human dermal fibroblast proliferation after seven days. In summary, the critical oxygen level seems to be around 2.0 bar and a repeated HBO treatment appears to be necessary for activating cellular signalling pathways, e.g. increased secretion of basic fibroblast growth factor and vascular endothelial growth factor as well as an inhibition of transforming growth factor-β1 [59].

Oxidative stress, production of ROS and their interaction with cellular proteins could induce apoptosis in inner hair cells and in SGN. Neurotrophic factors are essential for the survival of SGN and their withdrawal resulted in a complete apoptotic cell death of the SGN. Huang et al. (2000) [65] showed that BDNF withdrawal in spiral ganglion cell cultures resulted in higher levels of intracellular ROS in the neurons. For the present study, this means that the survival rate of SGN, which were cultivated with supernatants of HBO-treated cells (fibroblasts and MSCs), should be smaller if HBO treatment leads to a high ROS production. But it seems that this is not the case and this should be addressed in detail in further investigations. For HBO treatment, a clinical HBO protocol has been used and the duration of 90 min is rather short. Therefore, the antioxidant defences of the cells seemed to be sufficient, so that the induced biochemical stress, for example an increase in ROS formation, were almost completely reversible.

Finally, HBOT is an already accredited clinical therapy for sudden SNHL or barotrauma and could become a promising adjuvant treatment in association with cell-based therapies of the inner ear with immediate clinical translation. Therefore, HBO is a promising therapy for maintaining residual hearing in patients. Recently, Geng et al. [66] showed that the combination of transplanted MSCs and HBO treatment improved the recovery of lost hind limbs function in spinal cord-injured rats [66]. Based on these results, HBO may be used in conjunction with cochlear implantation with or without further biological therapies in order to improve the outcome. Thus, in order to advance this procedure towards clinical application, further studies determining the influence of HBO on cell-coated implants in vivo.

## Supporting information

S1 Fig BDNF-secretion per cell after HBO treatment. The mean BDNF concentrations were related to the corresponding mean of cell number. After one HBO treatment the HBO-treated NIH3T3/BDNF fibroblasts released 0.62 pg BDNF per cell and the NOR-treated cells released 0.64 pg/cells. After five treatments, the BDNF secretion per cell decreased to 0.52 pg per cell for the normoxic control and to 0.20 pg per cell for the HBO-treated fibroblasts. All values are given as mean ± standard error of the mean (SEM). Statistical analysis was performed by one-way ANOVA with Bonferroni’s multiple comparison test and was not significant.
(TIF)
