# Supplementary figures and images for: Effect of hyperbaric oxygen on BDNF-release and neuroprotection: Investigations with human mesenchymal stem cells and genetically modified NIH3T3 fibroblasts as putative cell therapeutics
Source: PLoS One. 2017 May 23;12(5):e0178182. doi: 10.1371/journal.pone.0178182 (PMC5441643; doi:10.1371/journal.pone.0178182)

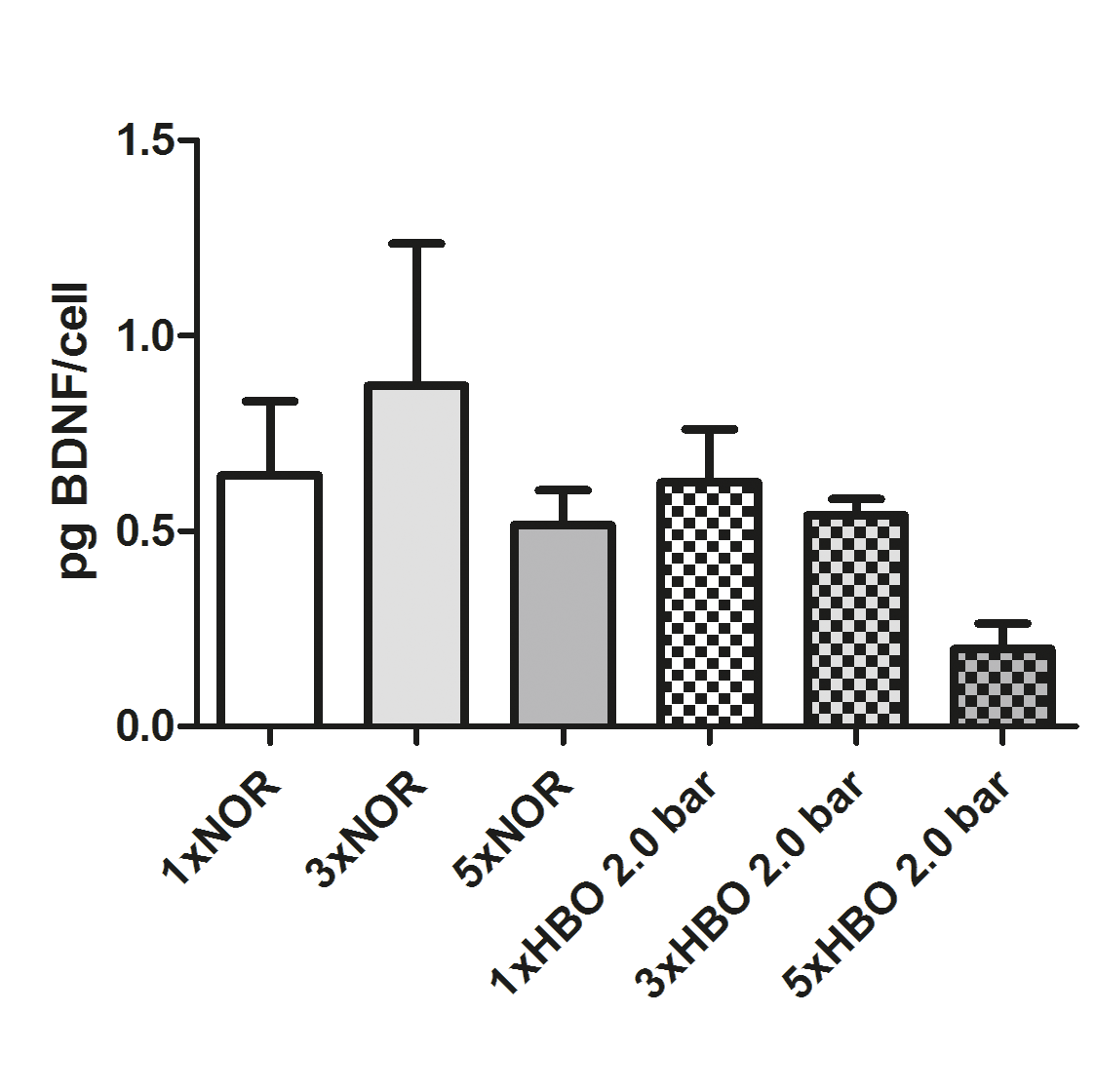

Supplement: S1 Fig — The mean BDNF concentrations were related to the corresponding mean of cell number. After one HBO treatment the HBO-treated NIH3T3/BDNF fibroblasts released 0.62 pg BDNF per cell and the NOR-treated cells released 0.64 pg/cells. After five treatments, the BDNF secretion per cell decreased to 0.52 pg per cell for the normoxic control and to 0.20 pg per cell for the HBO-treated fibroblasts. All values are given as mean ± standard error of the mean (SEM). Statistical analysis was performed by one-way ANOVA with Bonferroni’s multiple comparison test and was not significant. (TIF) [file pone.0178182.s001.tif]
